# Codon-Optimized RPGR Improves Stability and Efficacy of AAV8 Gene Therapy in Two Mouse Models of X-Linked Retinitis Pigmentosa

**DOI:** 10.1016/j.ymthe.2017.05.005

**Published:** 2017-05-24

**Authors:** M. Dominik Fischer, Michelle E. McClements, Cristina Martinez-Fernandez de la Camara, Julia-Sophia Bellingrath, Daniyar Dauletbekov, Simon C. Ramsden, Doron G. Hickey, Alun R. Barnard, Robert E. MacLaren

**Affiliations:** 1 Nuffield Laboratory of Ophthalmology, Department of Clinical Neurosciences, The John Radcliffe Hospital, Levels 5 & 6, West Wing, Headley Way, OX3 9DU Oxford, UK; 2 University Eye Hospital, Center for Opthalmology, Elfriede-Aulhorn-Strasse 7, 72076 Tübingen, Germany; 3 Centre for Genomic Medicine, Central Manchester University Hospitals NHS Foundation Trust, Manchester Academic Health Sciences Centre, M13 9WL Manchester, UK; 4 Oxford Eye Hospital, Oxford University Hospitals NHS Trust, The John Radcliffe Hospital, West Wing, Headley Way, OX3 9DU Oxford, UK

**Keywords:** gene therapy, retina, codon optimization

## Abstract

X-linked retinitis pigmentosa (XLRP) is generally a severe form of retinitis pigmentosa, a neurodegenerative, blinding disorder of the retina. 70% of XLRP cases are due to mutations in the retina-specific isoform of the gene encoding retinitis pigmentosa GTPase regulator (*RPGR*^*ORF15*^). Despite successful *RPGR*^*ORF15*^ gene replacement with adeno-associated viral (AAV) vectors being established in a number of animal models of XLRP, progression to human trials has not yet been possible. The inherent sequence instability in the purine-rich region of *RPGR*^*ORF15*^ (which contains highly repetitive nucleotide sequences) leads to unpredictable recombination errors during viral vector cloning. While deleted RPGR may show some efficacy in animal models, which have milder disease, the therapeutic effect of a mutated RPGR variant in patients with XLRP cannot be predicted. Here, we describe an optimized gene replacement therapy for human XLRP disease using an AAV8 vector that reliably and consistently produces the full-length correct RPGR protein. The glutamylation pattern in the RPGR protein derived from the codon-optimized sequence is indistinguishable from the wild-type variant, implying that codon optimization does not significantly alter post-translational modification. The codon-optimized sequence has superior stability and expression levels in vitro. Significantly, when delivered by AAV8 vector and driven by the rhodopsin kinase promoter, the codon-optimized RPGR rescues the disease phenotype in two relevant animal models (*Rpgr*^*−/y*^ and *C57BL/6J*^*Rd9/Boc*^) and shows good safety in *C57BL6/J* wild-type mice. This work provides the basis for clinical trial development to treat patients with XLRP caused by RPGR mutations.

## Teaser

X-linked retinitis pigmentosa caused by mutations in *RPGR* is a frequent cause of retinal degeneration and blindness without available treatment. Fischer et al. demonstrate safety and efficacy of gene supplementation in relevant animal models using a codon-optimized transgene, thereby resolving the problem of sequence instability of wild-type *RPGR*.

## Introduction

X-linked retinitis pigmentosa (XLRP) constitutes one of the most prevalent and devastating genetic disorders leading to blindness, with the majority caused by mutations in the *retinitis pigmentosa GTPase regulator* (*RPGR*) gene.^1^, ^2^, ^3^, ^4^ Previous work has demonstrated that relevant animal models could be rescued by expressing the wild-type (WT) cDNA of *RPGR*^*ORF15*^ in the affected photoreceptors.^5^, ^6^, ^7^, ^8^, ^9^, ^10^ However, the poor sequence stability of *RPGR*^*ORF15*^ as a transgene poses a significant challenge to maintaining sequence integrity during adeno-associated viral (AAV) production.^6^, ^8^, ^9^, ^10^ More worryingly, spontaneous mutations in the putative therapeutic transgene could potentially lead to a dominant-negative effect and accelerate disease progression.^11^ To date, the majority of research groups working in the field of RPGR gene therapy have independently seen inadvertent sequence modifications in the transgene during vector development.^5^, ^8^, ^9^, ^10^

A second challenge with developing *RPGR*^*ORF15*^ gene therapy is its complex posttranscriptional processing, which includes several splice variants.^12^ Because episomal AAV transgenes typically lack intronic sequences but encode the equivalent of mRNA with a polyadenylation signal, inadvertent processing (e.g., splicing) may occur on the “intronless” primary RNA transcript as it is exported from the nucleus. This may be even more likely in the case of *RPGR*^*ORF15*^, which contains many AG dinucleotides in its mutational hotspot, which could act as splice acceptors to many potential GT splice donor sites upstream. Furthermore, the open reading frame 15 (ORF15) region contains many single A nucleotide potential lariat branch points, and it should be noted that this part of the *RPGR* gene is spliced out in non-retinal tissues.

Another challenge of ocular gene therapy generally may be sub-therapeutic transgene expression after ocular delivery leading to limited and/or early loss of efficacy.^13^, ^14^ Potent *cis*-acting elements as well as intrinsic changes in the coding sequence (CDS) can help to improve efficiency of transduction without the need to increase the MOI. This is crucial as therapeutic transgene expression levels are achieved in the target tissue with minimal required number of viral vector particles, thereby reducing the risk of, e.g., capsid-related immunogenicity.^15^

We therefore decided to optimize the CDS of *RPGR*^*ORF15*^ to: (1) improve sequence stability, (2) remove cryptic splice sites, and (3) increase expression levels of the transgene. In vitro and in vivo data suggest that this provided us with a stable, safe, and efficacious vector for gene therapy in XLRP. We chose to test this approach in two relevant animal models, the targeted knockout *Rpgr*^*−/y*^ mouse line and *C57BL/6J*^*Rd9/Boc*^ mice, which carry a naturally occurring mutation in *Rpgr.*^16^, ^17^ Even though both animal models lack Rpgr^ORF15^ expression in the retina, both show a surprisingly mild phenotype with ∼20%–25% reduction in maximal b-wave amplitude at 6 months of age, which correlates closely to the observed outer nuclear layer (ONL) thickness loss.^17^ Interestingly, the slope of amplitude loss after the first 6 months follows that of aging wild-type *C57BL/6* mice.^17^ Because of this, we chose relatively large cohorts and limited ourselves to rescue of responses in electroretinography (ERG) as the efficacy endpoint to ensure sufficient statistical power. AAV8 was chosen as viral vector because of its transduction characteristics of primate photoreceptors following subretinal injection and its proven clinical safety profile.^18^, ^19^

## Results

### Codon-Optimized RPGR Shows a Higher Sequence Fidelity Than Wild-Type RPGR

The nucleotide sequence of *RPGR*^*ORF15*^ CDS was refined by reducing the frequency of low-abundance codons from 10% in the wild-type *RPGR*^*ORF15*^ (*wtRPGR*^*ORF15*^) to 1%, while increasing major codon usage from 32% to 56% (Figure S1). This improved the codon adaptation index (CAI) from 0.73 in the wild-type sequence to 0.87 for codon-optimized *RPGR*^*ORF15*^ (*coRPGR*^*ORF15*^). In addition to increasing the CAI, codon optimization also removed an *Mfe*I restriction site and several *cis*-acting elements such as a potential splice site (GGTGAT), four polyadenylation signals (three AATAAA and one ATTAAA), and two poly-T (TTTTTT) and one poly-A (AAAAAAA) sites. GC content and unfavorable peaks were optimized to prolong the half-life of the mRNA. Secondary structure formations (stem loops), which would reduce the chance of ribosomal binding and render mRNA less stable, were disabled. The pairwise identity between *wtRPGR*^*ORF15*^ and *coRPGR*^*ORF15*^ was 77.2%, with most changes introduced in the purine-rich region of the open reading frame 15.

The synthesized sequence of *coRPGR*^*ORF15*^ showed no sequence deviation throughout the steps toward successfully subcloning it into the plasmid for downstream recombinant AAV (rAAV) vector production. This is in contrast with the work with the plasmid containing *wtRPGR*^*ORF15*^, where several transformation experiments with XL10-Gold ultracompetent bacteria failed and, once successful, showed a 12 bp deletion in the ORF15 region in three independent samples (Figure S2). Sequencing the *wtRPGR*^*ORF15*^ construct at various stages of the subcloning posed a major challenge because of the repetitive nature and poly-G runs within the ORF15 region. Some regions required use of dGTP sequencing to improve read-through in purine-rich regions.

In eight independent cloning experiments (n = 4 for each construct), an average of 30 sequence runs were necessary to gain full coverage of wild-type construct, whereas a mean of 8 sequence runs was sufficient for a 2-fold coverage of the *coRPGR*^*ORF15*^ sequence. Alignment of sequence data to the reference (GeneID: 6103, isoform C) revealed numerous deletions, insertions, and point mutations of (mostly) single nucleotides in *wtRPGR*^*ORF15*^. In contrast, the integrity of the optimized sequence was maintained at all steps toward AAV production. Consequently, parameters of sequence fidelity, including the Phred quality scores Q20, Q30, and Q40 (Q20 indicates a base call accuracy of 99%, Q30 of 99.9%, and Q40 of 99.99%), mean confidence, and number of expected errors, were significantly weaker in Sanger sequencing data from *wtRPGR*^*ORF15*^ versus *coRPGR*^*ORF15*^ (Figure S3). Final proof for the superior sequence fidelity of *coRPGR*^*ORF15*^ was provided independently by the National Genetics Reference Laboratory (NGRL). After running 34 sequence reactions with *wtRPGR*^*ORF15*^ as a template, the cumulative data showed 74 ambiguous nucleotide calls (e.g., equal signal for guanine and adenine) and 6 potential insertion/deletion (in/del) mutations in the purine-rich ORF15 region. In contrast, complete coverage (two to seven times) was achieved using only half of the number of sequence reactions with the *coRPGR*^*ORF15*^ construct and not a single mutation was found (Figure S4).

### Increased RPGR Expression Levels through Codon Optimization

**Figure 1:**
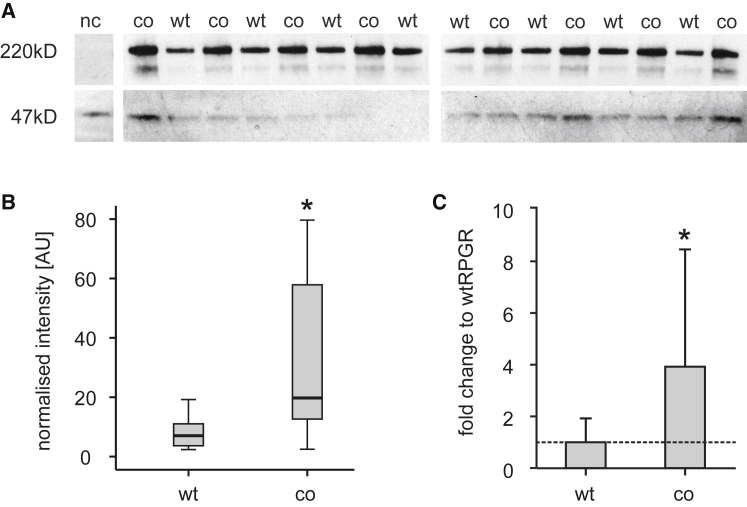
Analysis of RPGR^ORF15^ Expression Levels Based on Wild-Type versus Codon-Optimized Plasmid Constructs (A) Western blot of whole protein lysates from transfected HEK293T cells. Untransfected cells were used as negative control (nc), which only show a positive band at 47 kDa indicating the loading control GAPDH. (B and C) Codon-optimized and wild-type plasmid transfected cells were loaded in an alternating fashion, and signal intensity of bands at 220 kDa (indicating RPGR) were quantified. (B) Boxplot (median, box delineates lower and upper quartile, whiskers minimum and maximum) of intensities in arbitrary units (AU) after normalization to the loading control (GAPDH). (C) Bar graph (mean ± SD) after normalization to wild-type levels for a fold change presentation. After confirming the normal distribution of the dataset (n = 4), significance was tested by one-tailed t test for paired samples of unequal variance. *p < 0.005.

In order to analyze the effect of an increased CAI on the expression levels of RPGR^ORF15^, we compared wild-type and *coRPGR*^*ORF15*^ plasmid constructs head-to-head. Western blot analysis was used to assess expression levels in whole cell lysate from transfected HEK293T cells. Four independent six-well plate transfections, each with a technical replicate for wtRPGR and coRPGR, produced a total n of eight per construct (Figure 1). The *coRPGR*^*ORF15*^ construct showed approximately 4-fold higher expression levels (p = 0.01, t test). Fluorescence-activated cell sorting (FACS) was also used to compare expression levels of RPGR^ORF15^ in transfected HEK293T cells. Three independent experiments were conducted each with three technical replicates (n = 9) with HEK293T cells transfected with either *wtRPGR*^*ORF15*^, *coRPGR*^*ORF15*^, an *EGFP* construct (positive control), or media only (negative control [nc]). Positive controls showed *EGFP* expression at time of harvest, indicating that the transfection was successful and that the cells had enough time to produce a plasmid-encoded transgene. Cells transfected with the *coRPGR*^*ORF15*^ construct showed significantly higher protein expression levels (p < 0.01, Kruskal-Wallis test) compared with the cells transfected with the wild-type construct (Figure S5).

### Codon-Optimized RPGR CDS Translates into Full-Length RPGR Protein

**Figure 2:**
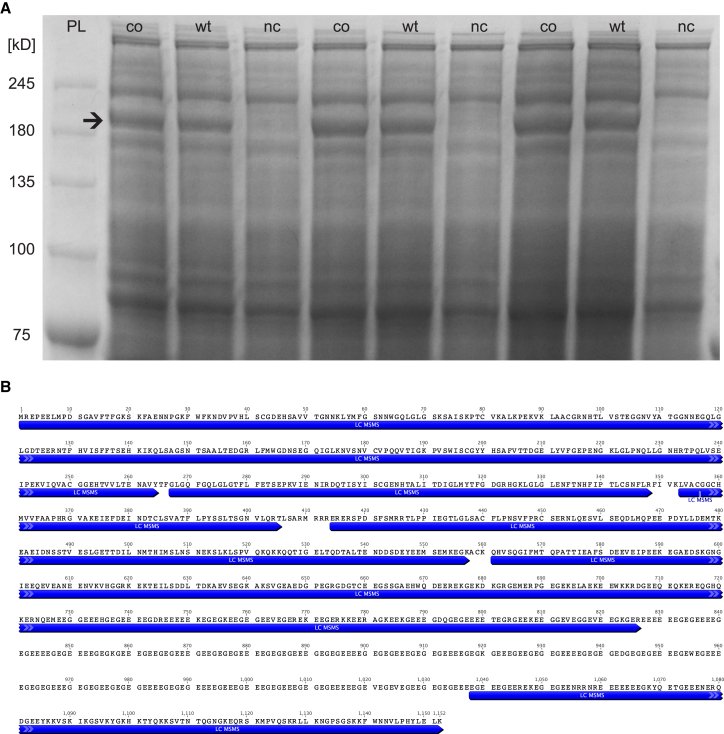
Liquid Chromatography-Tandem Mass Spectrometry of wtRPGR^ORF15^ and coRPGR^ORF15^ (A) Following overexpression in HEK293T cells, wtRPGR^ORF15^ and coRPGR^ORF15^ were purified from whole cell lysate by SDS-PAGE. In each lane, a single band above 180 kDa was identified as RPGR^ORF15^ (arrow). This band was missing in the negative control (nc) lanes. No additional bands are visible, which would be different between WT and coRPGR^ORF15^ lanes. (B) Identified RPGR^ORF15^ bands were then excised for liquid chromatography-tandem mass spectrometry (LC-MS/MS) analysis. More than 80% of peptide sequence could be directly identified (indicated by dark arrows below amino acid sequence). Unidentified amino acids (no arrows) are largely based in the ORF15 sequence, which escapes LC-MS/MS analysis because of its repetitive, glutamic acid and glycine-rich sequence. Note that the C-terminal sequence following the ORF15 region was identified, which rules out premature termination of translation. Together with the identical migration pattern between the wtRPGR^ORF15^ and coRPGR^ORF15^ samples in the SDS-PAGE (A), this suggests that codon optimization does not result in alternative splicing, but in a protein product equivalent to the wtRPGR^ORF15^.

To demonstrate that the substitution of nucleotides in the *RPGR*^*ORF15*^ does not lead to changes in the amino acid sequence, cryptic epitopes, or alternative peptide products, we analyzed recombinant RPGR^ORF15^ expression from WT and codon-optimized plasmids in HEK293T cells. Whole protein staining after SDS-PAGE demonstrated a single band difference between untransfected (nc) and transfected HEK293T cell lysates (Figure 2A). The single band was identical in size between cells transfected with either codon-optimized or WT plasmid sequences indicating no difference in migrating pattern between protein products derived from both CDSs, suggesting a similar, if not identical, combination of molecular weight, charge, and three-dimensional peptide structure. Bands were excised and analyzed by liquid chromatography-tandem mass spectrometry (LC-MS/MS) in order to determine the identity of the protein products derived from both expression plasmids. Both samples showed identical peptide sequences and confirmed the identity of the protein product of *coRPGR*^*ORF15*^ CDS as wtRPGR^ORF15^, also referred to as human RPGR isoform 6 (consensus CDSs CCDS35229.1). Due to the highly repetitive, glutamic-acid-rich ORF15 region, only ∼80% of all amino acids could be covered by the LC-MS/MS directly (Figure 2B). The lack of proteolytic target sites within ORF15 prevented the formation of oligopeptides of the “correct” size of 6–45 amino acids, which can be detected by LC-MS/MS. Instead either one large peptide of more than 200 amino acids would form, or cleavage between glutamic acid and glycine residues would result in peptide lengths below detection threshold of 6 amino acids. And even if these shorter sequences were detected, the highly repetitive nature of the ORF15 region would confound determining the precise origin of the peptide within the sequence. The fact that both plasmids resulted in an identical band on SDS-PAGE fits well with the LC-MS/MS evidence. Both suggest that the protein product derived from wild-type and codon-optimized RPGR CDS is identical to the reference sequence of RPGR^ORF15^. Even though some of the ORF15 sequence could not be verified by this approach, the intact C-terminal sequence from the LC-MS/MS experiment together with the correct band migration on SDS-PAGE suggest that *coRPGR*^*ORF15*^ translates into full-length wtRPGR^ORF15^.

### Post-translational Glutamylation of coRPGR^ORF15^ Is Indistinguishable from the Wild-Type

**Figure 3:**
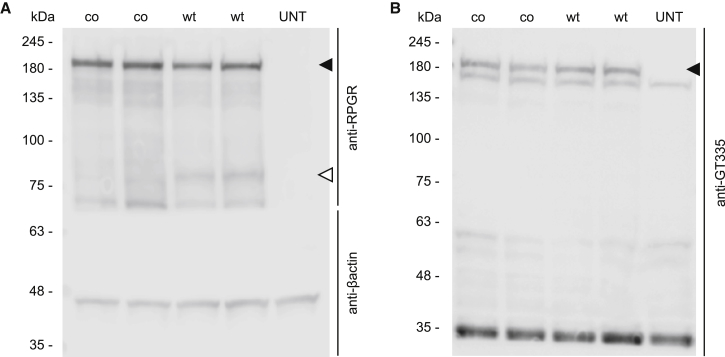
Western Blot Analysis of RPGR Expression and Glutamylation (A) RPGR^ORF15^ expression (black arrowhead) was detected in HEK293T cells transfected with either coRPGR^ORF15^ (co) or wtRPGR^ORF15^ (wt) containing plasmids compared with untransfected samples (UNT). A truncated 80 kDa protein (white arrowhead) was detected with an N terminus-directed RPGR antibody in cells transfected with the WT plasmid compared with cells transfected with the codon-optimized plasmid. (B) Glutamylated RPGR^ORF15^ was detected with the GT335 antibody in HEK293T cells transfected with the codon-optimized and the WT sequence of RPGR^ORF15^. (A and B) The 80 kDa band in (A) was not glutamylated in (B) and may therefore represent a truncated RPGR variant with a C-terminal deletion.

Because codon optimization may change the speed of translation, there have been concerns that this might also affect the post-translational modification of proteins. Glutamylation is an evolutionarily conserved post-translational modification that consists of the addition of glutamates to the C terminus of target proteins. Glutamylation was originally identified on the structural units of microtubules, α-tubulins, and β-tubulins, where it plays an important role regulating the interaction between microtubules and microtubule-associated proteins.^20^, ^21^ Glutamylation has recently been observed to be vitally important for RPGR^ORF15^ function,^22^, ^23^ and we were therefore interested to see whether this was altered following codon optimization. Western blot analysis on whole protein lysates showed a predominant band around 200 kDa, corresponding to RPGR^ORF15^ protein (Figure 3A) in HEK293T cells transfected with *coRPGR*^*ORF15*^ and *wtRPGR*^*ORF15*^ plasmids. Besides the expression of the full-length RPGR^ORF15^ sequences with both plasmid constructs, an additional band of 80 kDa molecular weight was detected in HEK293T cells transfected with the wild-type sequence (Figure 3A, white arrowhead). Glutamylation analysis in the same lysates revealed a GT335-immunoreactive band of the same molecular weight as the full-length RPGR protein (Figure 3B, black arrowhead). This result indicates that RPGR expressed by both constructs is glutamylated to a similar degree in vitro. The 80 kDa protein band seen only in the wild-type RPGR immunostaining (Figure 3A, white arrowhead) showed no glutamylation, in keeping with this being an aberrantly spliced RPGR variant with a large C-terminal deletion. Similarly, no glutamylated bands were seen in regions that did not correspond to bands on the RPGR western blot, hence it is likely that the RPGR antibody used has detected all RPGR variants that have an intact C terminus and ORF15. The absence of multiple bands of glutamylation indicated that the ORF15 region is being correctly translated without deletions and, therefore, confirms the correct protein sequence predicted by the LC-MS/MS analysis (Figure 2).

### Good Safety Profile of coRPGR^ORF15^ Transgene Expression

To test safety of the vector, 3-week-old wild-type mice (*C57BL/6J*) received subretinal injections of rAAV8 (1.5 × 10^9^ vector genomes [vg] in 1.5 μL) containing *coRPGR*^*ORF15*^ in a unilateral open-label trial (n = 19) and either *coRPGR*^*ORF15*^ or a non-coding control sequence as transgene in a bilateral, masked, controlled trial (n = 44). All mice recovered quickly after surgery and were subjected to ERG recordings at 2, 4, and 6 months of age. Ganzfeld ERG recordings showed no significant difference between either treated versus untreated (Figure S6) or treated versus sham-treated eyes (Figure S7) at any time point, suggesting that there was no major toxic effect originating from the surgery, recombinant AAV8, or the *coRPGR*^*ORF15*^ transgene. Retinal imaging with scanning laser ophthalmoscopy (SLO) also showed no damage to ocular structures after subretinal application of rAAV8.coRPGR^ORF15^, supporting the view of a safe application (Figure S8).

### Codon-Optimized RPGR^ORF15^ Rescues Disease Phenotype

**Figure 4:**
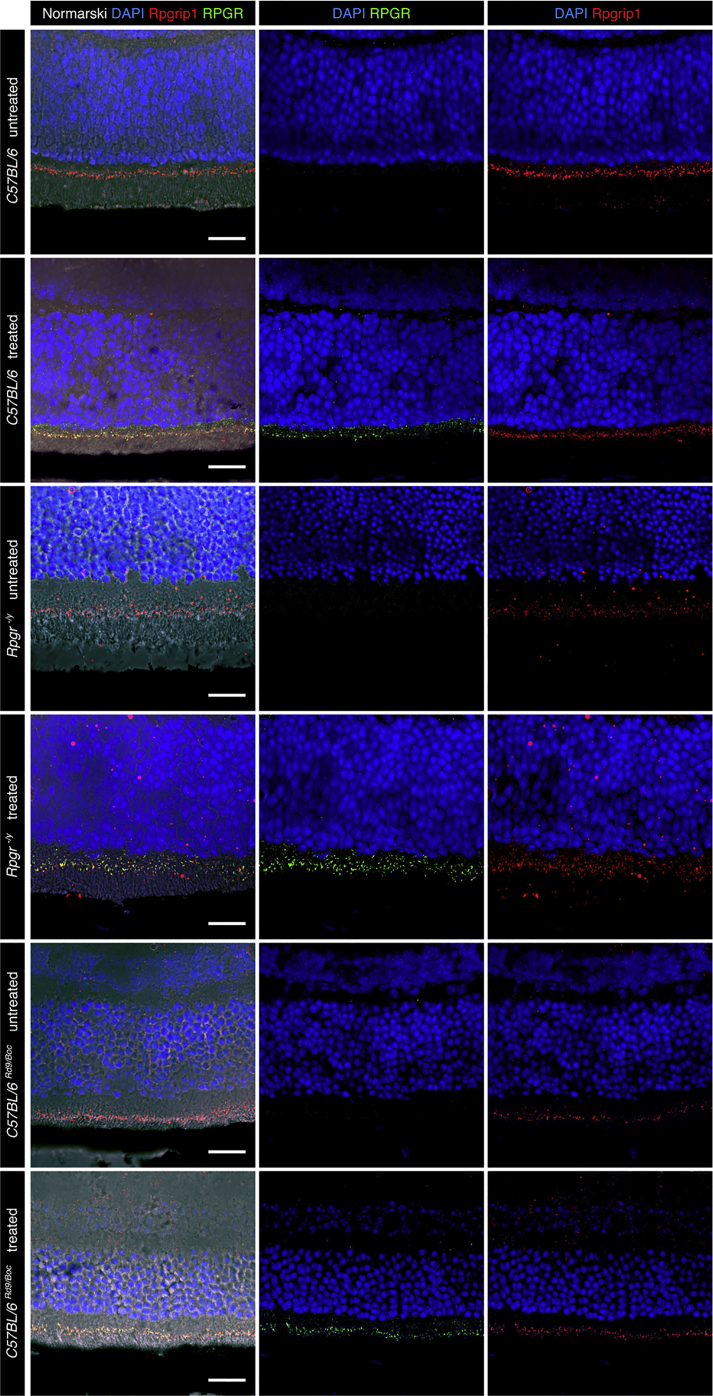
Immunohistochemical Analysis of Treated and Untreated Eyes of *C57BL/6*, *C57BL/6*^*Rd9/Boc*^, and *Rpgr*^*−/y*^ mice Representative sections from control untreated (top panels) and AAV.*coRPGR*^*ORF15*^-treated eyes in *C57BL/6* mice were stained with Hoechst (blue) and antibodies against human RPGR (SIGMA N-terminal; green) and mouse Rpgrip1 (red). Top panels show no human RPGR expression in a control untreated eye, whereas Rpgrip1 (red) indicates location of the connecting cilia. Treatment with AAV.*coRPGR*^*ORF15*^ resulted in RPGR expression (second row) and co-localization of human RPGR with Rpgrip1 (red). Middle panels show the same for *C57BL/6*^*Rd9/Boc*^ mice and lower panels for *Rpgr*^*−/y*^ mice. Scale bars, 20 μm.

**Figure 5:**
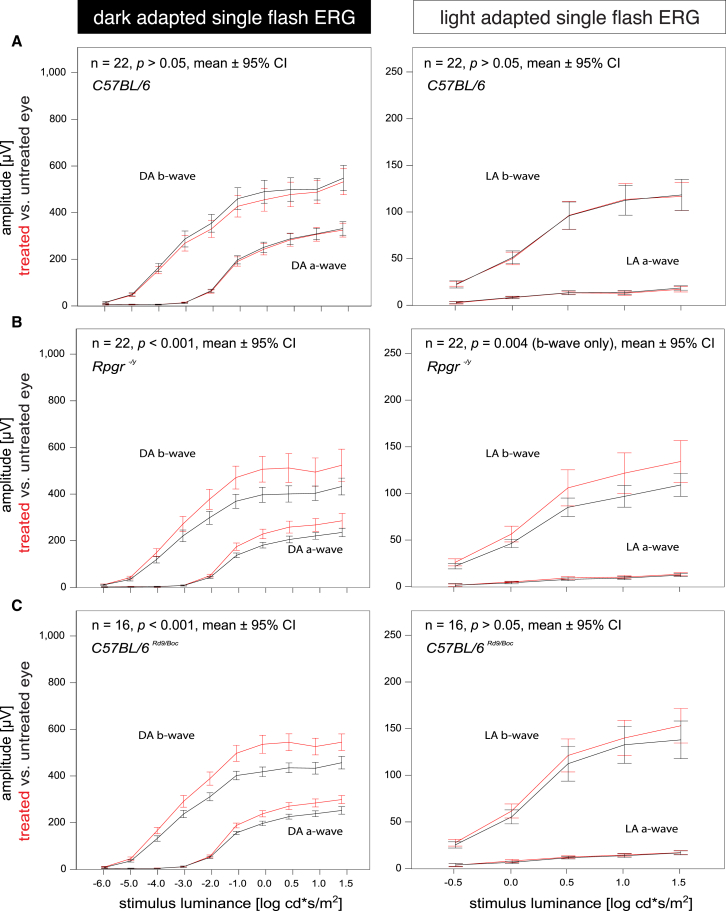
Electroretinographic Analysis of Treated versus Untreated Eyes of *C57BL/6*, *Rpgr*^*−/y*^, and *C57BL/6*^*Rd9/Boc*^ Mice at 6 Months of Age Left panels indicate mean (±95% confidence interval) amplitudes of dark-adapted a- and b-wave amplitudes after single light flashes over a luminance series. Right panels show results in the light-adapted state. (A–C) Lack of toxicity in *C57BL/6* mice is shown in (A), whereas sum potentials show treatment effects in both disease models *Rpgr*^*−/y*^ (B) and *C57BL/6*^*Rd9/Boc*^ mice (C). The most robust treatment effect can be observed between −1 and 1 log cd*s/m^2^ in the dark-adapted luminance series. Red represents treated eyes; black represents untreated eyes.

Three-week-old *C57BL/6J*^*Rd9/Boc*^ and *Rpgr*^*−/y*^ mice were treated with unilateral (n_Rd9_ = 18, n_ko_ = 24) or bilateral (treatment versus sham, n_Rd9_ = 17, n_ko_ = 20) injections (1.5 × 10^9^ vg in 1.5 μL). Immunohistochemistry of unfixed cryosections showed that the antibody detecting human RPGR does not cross-react with the orthologous Rpgr in *C57BL/6J* wild-type mice (Figure 4). More importantly, it demonstrated RPGR transgene expression and co-localization with the ciliary protein Rpgrip1 in the treated eye of *C57BL/6J*^*Rd9/Boc*^ and *Rpgr*^*−/y*^ mice, but not the control eye. In the longitudinal ERG analyses, both animal models of XLRP showed significant rescue of the dominating rod system at 6 months, the last time point studied (Figure 5). *Rpgr*^*−/y*^ mice also showed a significant rescue of cone function under light-adapted conditions, whereas the responses in *C57BL/6J*^*Rd9/Boc*^ mice showed a trend for efficacy, which did not reach the significance level in light-adapted conditions at this time point. Some of the measures at earlier time points also showed significant therapeutic effects or a trend for efficacy again especially at higher stimulus intensities (Figures S9 and S10). It is important to note that ERG responses from treated eyes versus untreated eyes (intra-individual comparison) showed significant rescue with the therapeutic AAV vector in both animal models at 4 and 6 months of age. However, when mice were treated with the therapeutic vector in one eye and the control vector in the other, variability increased as a result of having surgery performed on both sides. This contributed to variability in the ERG signals. Importantly, all responses from the treated eyes were still always greater than those from the control treated eyes. However, the significance threshold was only met in *Rpgr*^*−/y*^ mice at 6 months.

## Discussion

*RPGR* replacement therapy by AAV has been a goal for the scientific community since the characterization of *RPGR* as the genetic cause for XLRP.^1^, ^24^ The fact that it is still a goal that has not translated into a clinical trial is mainly due to the fact that *RPGR* is a complex gene with high propensity for mutational changes.^12^, ^25^ This has caused delays in the development of appropriate datasets for the support of investigational new drug applications. Even with regulatory approval for a phase I trial, production of clinical grade AAV for RPGR gene therapy is associated with significant risks because the *RPGR* transgene might spontaneously mutate in the course of vector production according to good manufacturing practice (GMP) guidelines.

Here we present evidence regarding the safety and efficacy of a new type of vector construct for RPGR gene therapy in wild-type mice (*C57BL/6J*) and two relevant animal models (*Rpgr*^*−/y*^ and *C57BL/6J*^*Rd9/Boc*^ mice). This rAAV vector features a codon-optimized CDS of *RPGR*^*ORF15*^. Evidence is provided suggesting superior sequence stability, easier sequence analysis, reduced risk of cryptic epitopes/unwanted splice variants, and increased expression levels for the codon-optimized CDS of *RPGR*^*ORF15*^. Proteomic analysis demonstrates that the resulting peptides from both wild-type and *coRPGR*^*ORF15*^ display the same biochemical and sequence characteristics. Immunohistochemistry and functional data from animal models also suggest that the resulting peptide localizes to its physiological cellular compartment, the connecting cilium, and protects against loss of retinal function because of lack of *Rpgr* expression. The ORF15 region in the C terminus of RPGR protein contains 11 glutamate-rich consensus motifs.^22^ The addition of negatively charged glutamates to this region could affect the stabilization and the folding of RPGR protein and its interaction with other proteins in the connecting cilium. The absence of this modification could therefore affect the binding of RPGR to transport and scaffold proteins, compromising RPGR function.^23^ In this study, we show that RPGR protein is expressed by both constructs tested (codon-optimized and wild-type sequences) and it is glutamylated in a human cell line, suggesting its correct post-transcriptional processing following plasmid transfection. Importantly, the present evidence shows that a single subretinal application of AAV.*coRPGR*^*ORF15*^ is safe and/or may stop or slow down the functional loss linked to retinal degeneration in *Rpgr*^*−/y*^ and *C57BL/6J*^*Rd9/Boc*^ mice.

To explore potential toxic effects of AAV.coRPGR, we tested 1.5 × 10^9^ vg in 63 wild-type *C57BL/6J* mice, but found no significant differences between measures of retinal function in ERG at any time point between the treatment group and the sham or untreated group (Figures S6 and S7). Additionally, in vivo retinal imaging suggests that there was no impact of AAV.*coRPGR*^*ORF15*^ treatment on retinal structure. Immunohistochemistry demonstrated RPGR^ORF15^ expression and co-localization with ciliary protein Rpgrip1 in treated animals, but not in untreated or control treated animals (Figure 4). Taken together, single treatment of wild-type mice with AAV.*coRPGR*^*ORF15*^ did not seem to induce any toxic effects and showed a good safety profile. This is in line with similar reports from the literature, where different constructs also led to expression of wild-type or variants of RPGR^ORF15^ in wild-type mice.^8^, ^9^ Toxic effects could principally be observed with some truncated versions of Rpgr or with overexpression of RPGR.^8^, ^9^, ^11^ We did not see any negative effects in the current trial, and therefore conclude that treatment with AAV.*coRPGR*^*ORF15*^ at the chosen dose is safe.

The therapeutic effect of AAV.*coRPGR*^*ORF15*^ was demonstrated in two well-characterized mouse models of XLRP caused by *Rpgr* mutations: the targeted knockout model *Rpgr*^*−/y*^ (kindly provided by Tiansen Li) and *C57BL/6J*^*Rd9/Boc*^ featuring a naturally occurring mutation in *Rpgr*, the murine ortholog of *RPGR*.^16^, ^17^ Both models have been shown to lack Rpgr^ORF15^ expression in the retina, and hence were chosen as relevant animal models. However, there are some caveats with using these animal models. Most importantly, the disease phenotype is surprisingly mild, which necessitates relatively large cohorts in a trial to gain the necessary statistical power. As a consequence, we conducted trials in a total of ∼80 animals and provided evidence of efficacy as indicated by significant rescue of electrophysiological measurements in *Rpgr*^*−/y*^ and *C57BL/6J*^*Rd9/Boc*^ mice. The treatment did not become evident at the first time point (i.e., 2 months of age) most likely because of the slow disease progression in both animal models. However, AAV.*coRPGR*^*ORF15*^ treatment was associated with significant ERG rescue in both animal models at postnatal month (PM)4 and PM6. This rescue was more evident in the dark-adapted luminance series, which reflects the sum potential of rod photoreceptor pathway in the lower luminance range (single flashes up to ∼0.01 cd*s/m^2^).^26^ Stimuli with higher flash luminance are thought to stimulate a mixed cone-rod pathway response. The biggest difference between the treated and sham-treated/untreated eyes were seen at a luminance of around 1 cd*s/m, which likely results from a mixed rod-cone pathway response. A temporary treatment effect of AAV.*coRPGR*^*ORF15*^ seems unlikely in view of the persistent effects in the dark-adapted luminance series. Also, previous studies have indicated that RPGR expression in *Rpgr*^*−/y*^ mice leads to sustained treatment effects.^8^, ^9^ The discrepancy between the unilateral, open-label experiments and the bilateral, sham-controlled experiments may indicate the possibility that the sham treatment also had a mild effect on ERG. There is some evidence in other animal models that surgical intervention in the eye induces a therapeutic effect.^27^ This is thought to be initiated by injury-induced paracrine release of neurotrophic factors, e.g., from activated glia cells.^28^ Although we cannot completely rule out a mild sham effect, there were significant differences between AAV.*coRPGR*^*ORF15*^-treated and sham-treated eyes in the dark- and light-adapted ERG responses, e.g., in *Rpgr*^*−/y*^ mice at different time points.

Only one vector dose (1.5 × 10^9^ vg) was tested in this study, which addressed the question whether the chosen dose would rescue the ERG in relevant animal models of XLRP and whether this dose would have a toxic effect in wild-type. A formal toxicology study according to good laboratory practice (GLP) guidelines would need to test multiple doses to provide a complete vector safety profile. However, although this may give some indication of the therapeutic window, the maximally tolerated dose in humans would still have to be determined in a clinical trial.

Efficacy of treatment was defined as functional rescue with ERG as an important, objective outcome measure of retinal function that has been shown to correlate closely with ONL loss in mice lacking Rpgr^ORF15^ expression.^17^ However, ERG can also be influenced, e.g., by length of photoreceptor outer segments, and thus only indirectly reflects preservation of photoreceptors. Because eyes were processed to study RPGR expression and localization as a structural marker of efficacy, unfixed retinal cryosections were obtained to ensure epitope recognition in the connecting cilium. However, such unfixed sections were not considered appropriate for ONL thickness measurements, another potential structural endpoint for efficacy.

Taken together, treatment with AAV.*coRPGR*^*ORF15*^ appears to be safe and effective. Successful transduction of photoreceptors with AAV.*coRPGR*^*ORF15*^ in wild-type mice did not lead to toxic effects, which might have been associated with the expression of RPGR^ORF15^ on a background of physiological levels of native Rpgr. Furthermore, treatment of animal models of XLRP showed a statistically significant rescue of ERG responses in the treated eyes, but not in the untreated eyes. These data are the first evidence that a codon-optimized CDS of *RPGR*^*ORF15*^ can be used to safely treat eyes that lack expression of this important ciliary protein. These findings have recently been translated into a Phase I/II gene therapy clinical trial to treat XLRP in humans (NCT03116113). Orphan drug programs with the European Medicines Agency and Food and Drug Administration should allow for a fairly rapid process of developing a first treatment for this devastating blinding disorder.

## Materials and Methods

### Codon Optimization

Geneious software (v6.1.6; Biomatters) was used to search the consensus CDS (CCDS) database of the NCBI for the reference human *RPGR*^*ORF15*^ nucleotide sequence. The complete CDS was subjected to the OptimumGene algorithm (GenScript) to optimize a variety of parameters that are critical to the efficiency of gene expression, including codon usage bias, GC content, CpG dinucleotides content, mRNA secondary structure, cryptic splicing sites, premature poly-A sites, internal chi sites and ribosomal binding sites, negative CpG islands, adenylate-uridylate-rich elements (AREs), repeat sequences (direct repeat, reverse repeat, and dyad repeat), and restriction sites that may interfere with cloning.

### Viral Vector Production

AAV2/8 batches were produced by transient co-transfection of HEK293T cells seeded in cell factories (HYPERflask; Corning), purified using iodixanol gradient ultracentrifugation, and concentrated by buffer exchange (Amicon Ultra-15; Millipore) to remove residual iodixanol as previously described.^29^ Capsid proteins for the AAV2/8 were encoded by helper plasmid pDP8.ape (PlasmidFactory). The commercial plasmid AAV2-CAG-GFP (cat. no. 7072; Vector Biolabs) was used to provide the virus genome with AAV2 inverted terminal repeat (ITR) sequences flanking the CAG-GFP CDS and the bovine growth hormone poly-A (bGHpA) signal targeted for RT-PCR-based quantification of vg. The photoreceptor-specific human rhodopsin kinase (RK) promoter was used upstream of the RPGR^ORF15^ sequence to replace the CAG-GFP CDS and in order to restrict the expression of the transgene to the photoreceptors.

### Titer Quantification

Viral capsids were subjected to DNase treatment and denatured at 95°C to release vg for PCR amplification using a CFX Connect RT-PCR system (Bio-Rad) as described previously.^30^ A standard curve was derived from a DNA plasmid of known concentration containing the same bGHpA sequence that was targeted by the primer pair used for the samples (forward: 5′-CCAGCCATCTGTTGTTTGCC-3′, reverse: 5′-GAAAGGACAGTGGGAGTGGC-3′. Cycle threshold (Ct) detection and the standard curve were calculated using the default settings of the CFX Manager (version 3.0; Bio-Rad) RT-PCR software and enabled to calculate the vg concentration in test samples. Each sample was tested in triplicate.

### Animal Experimentation

All animals received 1.5 × 10^9^ vg in 1.5 μL via the NanoFil subretinal injection kit (WPI) equipped with a 34-gauge needle with a 25° tri-surface bevel optimized for microinjection. Injection into the subretinal space was achieved under direct visual guidance, ab externo and posterior to the equator as described elsewhere.^31^ For subretinal injections and in vivo imaging and ERG, animals were anesthetized with ketamine (80 mg/kg) and xylazine (10 mg/kg). Imaging was performed as previously described using a confocal scanning laser ophthalmoscope (Heidelberg Engineering).^32^, ^33^ ERG was performed using an Espion E2 system (Diagnosys) as previously described.^34^ For dark-adapted testing, responses were elicited by brief flashes of white light recorded on a dark background. Stimulus luminance was increased in log unit steps across a 7 log unit range (−6 to 1 log cd*s/m^2^). At each luminance tested, up to 16 responses were averaged per result. An interstimulus interval (ISI) of 3 s was used for dimmer stimuli (−6 to −3 log cd*s/m^2^), and for brighter luminance (−2 to 1 log cd*s/m^2^) an increasing ISI of 5–30 s was used. For light- adapted testing, animals were pre-exposed to a steady, full-field, white background (30 cd/m^2^) for 10 min. Responses were then recorded to brief light flashes with four stepwise increases in luminance (−0.52, 0, 0.48, 1, and 1.4 log cd*s/m^2^) superimposed on the stable background. In all cases, an ISI of 1 s was used and 20 responses were averaged per result. At the end of the in-life phase, animals were sacrificed and eyes processed for immunohistochemistry as previously described.^35^

### Western Blot

HEK293T cells were harvested 48 hours after plasmid transfection, and protein was extracted with radio immunoprecipitation assay (RIPA) lysis buffer as previously described.^36^ Supernatant was quantified using the Pierce Bicinchoninic Acid (BCA) Protein Assay Kit (Thermo Scientific), and samples were denatured in Laemmli buffer (Sigma-Aldrich) for 20 min at room temperature. Ten micrograms total protein was loaded per well using 7.5% SDS polyacrylamide gels (Criterion TGX Precast Gels; Bio-Rad Laboratories) for electrophoresis at 100 V for 2 hr (SDS-PAGE). Proteins were blotted onto polyvinylidene difluoride (PVDF) membranes with 0.2 μm pore size (Trans-Blot Turbo Midi PVDF; Bio-Rad) using the Trans-Blot Turbo Transfer Starter System (Bio-Rad) using the midi setting (7 min at 25 V). PVDF membranes were then cut into sections depending on the size of target protein and loading control to stain independently with respective primary antibodies (anti-RPGR [N-terminal, rabbit polyclonal at 1:500; Cat No. HPA001593; Sigma-Aldrich] and anti-glyceraldehyde 3-phosphate dehydrogenase [GAPDH] [mouse monoclonal at 1:2000; cat. no. TA802519; OriGene]), and horseradish peroxidase (HRP)-linked secondary antibodies (1:10,000; cat. no. ab16284 and ab6820; Abcam) were used for detection by chemiluminescence with Luminata forte ELISA HRP substrate (Millipore). Membrane sections were recorded with the Odyssey imaging system (LI-COR Biosciences) and analyzed with the Image Studio Lite software, version 5.0.

### LC-MS/MS

Protein samples of plasmid (containing either *wtRPGR* or *coRPGR*) transfected HEK293T cells were processed as described above for SDS-PAGE. EZBlue Gel Staining Reagent (Sigma-Aldrich) was then used to stain proteins according to the manufacturer’s instructions: the SDS-PAGE gel was rinsed three times for 5 min each in an excess of water to remove SDS before incubating the gel in the EZBlue Gel Staining Reagent for 2 hr at room temperature on a shaker. The gel was then washed in excess water for 2 hr before an image was taken and the appropriate bands (containing either wtRPGR or coRPGR protein) excised with a disposable scalpel. Bands were stored in 1.5 mL Eppendorf tubes at 4°C until further processing at the Proteomics Centre of the University of Oxford (Dunn School of Pathology). Samples were digested using trypsin, lysine C, lysine N, pepsin, formic acid, elastase, and/or V8 protease followed by LC-MS/MS. Peptide fragments were recorded along with their sequence identity and matched to the human proteome.

### Glutamylation Pattern of RPGR

Glutamylation was assessed by western blot analysis using the GT335 antibody (mouse monoclonal at 1:500; cat. no. AG-20B-0020-C100; AdipoGen). To identify the comigration of GT335-reactive bands with human RPGR, we used the same RPGR antibody raised against the N terminus (see Materials and Methods for western blot) from Sigma-Aldrich. Mouse monoclonal anti-β-actin antibody at 1:30,000 (cat. no. AM4302; Thermo Scientific) was used as loading control. A total of 60 μg of total protein was denatured and processed as described above for western blot. The membranes were blocked with 3% BSA in PBS containing 0.1% Tween 20 for 45 min and incubated with primary antibodies at room temperature for 1 hr. Bands were detected with HRP-conjugated secondary antibodies with the use of enhanced chemiluminescence (ECL) detection reagent (Bio-Rad) using the Odyssey imaging system (LI-COR Biosciences) and analyzed with the Image Studio Lite software, version 5.0.

### Immunohistochemistry and Flow Cytometric Analysis

For immunohistochemistry of treated or untreated mouse eyes, only unfixed tissue was used in order to detect ciliary proteins, such as RPGR^ORF15^, because antigen detection at the connecting cilium is hampered by the cross-linking activity of fixatives such as formaldehyde. Eyes were collected as described above, quickly embedded in Tissue-Tek O.C.T. Compound (Sakura Finetek USA) without fixation and/or dehydration, and frozen in dry ice cooled isopentane. Samples were stored at −80°C until cryosections were prepared at 16 μm thickness and collected on a poly-L-lysine-coated glass slide (Gerhard Menzel). Following a brief wash with 0.01 M PBS (1 min), sections were blocked with 10% donkey serum in PBS containing 2% BSA for 10 min and then incubated with primary antibody diluted in 2% BSA for 45 min. Primary antibodies were directed against the N terminus of RPGR (rabbit polyclonal at 1:200; cat. no. HPA001593; Sigma-Aldrich) against Rpgrip1 (goat polyclonal [E14] at 1:200; cat. no. sc-390330; Santa Cruz), and secondary antibodies were donkey anti-rabbit Alexa Fluor 488 at 1:5,000 (cat. no. A-31573; Thermo Fisher) and donkey anti-goat Alexa Fluor 647 at 1:5,000 (cat. no. A-21447; Thermo Fisher). Primary and secondary antibodies were diluted in 0.01 M PBS with 0.1% Triton X-100 and 1% BSA. Secondary antibody solution also contained Hoechst 33342 dye at 1:5,000. Sections were mounted in ProLong Gold (Life Technologies) for fluorescence microscopy only minutes to hours later on a confocal microscope (Zeiss LSM710).

For flow cytometric analysis, plasmid transfected HEK293T cells were labeled for RPGR with the same primary antibody directed against the N terminus of RPGR described above (Sigma-Aldrich) and a secondary antibody with conjugated fluorescent dye (donkey anti-rabbit Alexa Fluor 647 at 1:5,000; cat. no. A-31573; Thermo Fisher). Primary and secondary antibodies were diluted in 0.01 M PBS with 0.1% Triton X-100 and 1% BSA. In brief, 48 hours after transfection, cells were washed before resuspension to approximately 1–5 × 10^6^ cells/mL in ice-cold 0.01 M PBS. After fixation in 1% (v/v) paraformaldehyde (PFA) for 10 min at 4°C, cells were gently pelleted down at 120 × *g* for 5 min at 4°C. Aqueous solution was carefully aspirated and cells resuspended in blocking solution (10% [w/v] donkey serum in PBS-T [0.1% Triton X-100 in 0.01 M PBS]). After 30 min, cells were spun again as above and supernatant removed. Primary antibody solution was added at the appropriate concentration, and the sample was incubated at room temperature for 2 hr. After three wash steps (cells pelleted down at 120 × *g* for 5 min at 4°C, supernatant removed, cells resuspended in ice-cold PBS-T), the secondary antibody was added for 30 min in the dark at room temperature, followed by the same washing procedure. Cells were kept on ice until further processing (within 1 hr). Cell suspension was subjected to flow cytometry using a CyAn Advanced Digital Processing (ADP) LX High-Performance Research Flow Cytometer (DakoCytomation; Beckman Coulter) at the Flow Cytometry Facility of the University of Oxford (The Jenner Institute, Nuffield Department of Medicine). Gate settings were chosen for a false discovery rate of <1% for quantitative analysis of positive cells and their median fluorescence intensity.

### Statistical Analysis

Data were analyzed using Statistical Package for Social Sciences (SPSS) version 21 by IBM (SPSS, version 21; IBM) for macOS. ERG data were exported as tab delimited text files and imported into Excel (v14.1.0; Microsoft). Custom macros were written in Visual Basic to automate extraction of a- and b-wave amplitudes for single flash recordings. These data were then imported for statistical analysis into SPSS. The false discovery rate associated with multiple testing of ERG data was controlled for by the False Discovery method published by Benjamini et al.^37^ Data were tested for normality of distribution using the Shapiro-Wilk test. If the null hypothesis of normality was retained, ANOVA or a Student t test was used to test for a significant difference where appropriate. Nonparametric tests such as the Kruskal-Wallis or Mann-Whitney *U* test were otherwise applied as appropriate. Statistical significance was defined as α = 0.05 for all tests. Data are shown as mean and SEM unless stated otherwise.

## Author Contributions

M.D.F. planned and executed experiments, analyzed data, and wrote the manuscript. M.E.M. planned and supervised experiments, helped to interpret data, and edited the manuscript. C.M.-F.d.l.C. planned and executed experiments and analyzed data. J.-S.B. planned and executed experiments. D.D. and D.G.H. assisted in experiments and critiqued analysis. A.R.B. helped to interpret data, critiqued analysis, and edited the manuscript. R.E.M. planned experiments, helped to interpret data, critiqued analysis, and edited the manuscript.

## Conflicts of Interest

R.E.M. is the academic founder and a director of NightstaRx Ltd., a gene therapy company established by the University of Oxford and funded by the Wellcome Trust. M.D.F. and R.E.M. are named inventors on a patent filed on behalf of the University of Oxford, relating to the expression cassette and codon optimization of RPGR coding sequence in general. R.E.M., M.D.F., and A.R.B. are consultants to NightstaRx Ltd. R.E.M. and M.D.F. have received grant funding to their academic institutions from NightstaRx Ltd. No competing financial interests exist for M.E.M., C.M.-F.d.l.C., J.-S.B., D.D., S.C.R., or D.G.H.

## Supplemental Information

Document S1. Figures S1–S10

Document S2. Article plus Supplemental Information
