# Supplementary material for: Codon-Optimized RPGR Improves Stability and Efficacy of AAV8 Gene Therapy in Two Mouse Models of X-Linked Retinitis Pigmentosa
Source: Mol Ther. 2017 May 24;25(8):1854–65. doi: 10.1016/j.ymthe.2017.05.005 (PMC5542800; doi:10.1016/j.ymthe.2017.05.005)
Supplement: Document S1. Figures S1–S10 [file mmc1.pdf]

YMTHE, Volume *25*

Supplemental Information

Codon-Optimized RPGR Improves Stability and Efficacy of AAV8 Gene Therapy in Two Mouse Models of X-Linked Retinitis Pigmentosa

M. Dominik Fischer, Michelle E. McClements, Cristina Martinez-Fernandez de la Camara, Julia-Sophia Bellingrath, Daniyar Dauletbekov, Simon C. Ramsden, Doron G. Hickey, Alun R. Barnard, and Robert E. MacLaren

Supplemental Materials

Fig. S1. Overview over wild-type *RPGR* at Xp11.4 and the changes to optimise the coding sequence of $RPGR^{ORF15}$.

Fig. S2. Sequencing of the $wtRPGR^{ORF15}$ cloning vector.

Fig. S3. Superior sequence fidelity of $coRPGR^{ORF15}$ over $wtRPGR^{ORF15}$.

Fig. S4. Independent confirmation of superior sequence stability of $coRPGR^{ORF15}$.

Fig. S5. Flow cytometric analysis of $\mathrm{RPGR}^{\mathrm{ORF15}}$ expression.

Fig. S6. RPGR gene therapy shows no toxicity in electroretinography (ERG) of unilaterally treated wild-type mice.

Fig. S7. RPGR gene therapy shows no toxicity in electroretinography (ERG) of bilaterally treated wild-type mice.

Fig. S8. Representative retinal images of *C57BL/6J* mice at postnatal month 6 (PM6).

Fig. S9. ERG recordings in $Rpgr^{-/y}$ mice after $coRPGR^{ORF15}$ gene therapy.

Fig. S10. ERG recordings in $C57BL/6J^{Rd9/Boc}$ mice after $coRPGR^{ORF15}$ gene therapy.

Supplemental Figure Legends:

S1. Overview over wild-type *RPGR* at Xp11.4 and the changes to optimise the coding sequence of $RPGR^{ORF15}$. (**A**) $\text{RPGR}^{\text{ORF15}}$ is the longest RPGR isoform (consensus coding sequences CCDS35229.1) and encodes for an 1152 amino acid protein with distinct domains. The more N-terminal RCC1-like domain is common to all known RPGR isoforms, while the glycine/glutamic acid rich domain and the carboxy-terminus are unique for $\text{RPGR}^{\text{ORF15}}$. (**B**) Codon optimisation of $RPGR^{ORF15}$ leads to significant changes in the primary coding sequence. Here, altered GC frequency (%) is indicated along the full coding sequence of $RPGR^{ORF15}$ with wild-type $RPGR^{ORF15}$ indicated on the top (black) and codon optimised $RPGR^{ORF15}$ ($coRPGR^{ORF15}$) at the bottom (red) with grey breaks indicating the changes from the wild-type sequence. (**C**) The full sequence is displayed with $coRPGR^{ORF15}$ on top (Optimized) indicating the silent substitutions indicated in red, while the wild-type $RPGR^{ORF15}$ sequence is displayed as reference below (Original).

S2. Sequencing of the $wtRPGR^{ORF15}$ containing cloning vector revealed a 12bp deletion in the ORF15 region (c.3052_3063del), which would lead to an in-frame loss of four aminoacids (Gly-Arg-Gly-Ser).

S3. Superior sequence fidelity of $coRPGR^{ORF15}$ over $wtRPGR^{ORF15}$. (**A**) While all $\text{wtRPGR}^{\text{ORF15}}$ plasmid preparations featured at least some mutations, none of the $coRPGR^{ORF15}$ plasmid preparations was found to harbour any deletion, insertional- or point mutation. (**B**) Percentage of nucleotides within each plasmid sequence with at least 99% (first row), 99.9% (second row) or 99.99% (third row) base call accuracy. These levels of confidence of individual base calls corresponds to the Phred quality scores Q20, Q30 and Q40 respectively. Numbers are reported as mean ± standard deviation. Mean confidence level of base call accuracy (fourth row) and number of expected errors (bottom row) for each plasmid sequence. Numbers are reported as mean ± standard deviation. Statistical analysis was performed with Student t-test (n=4) and corrected for multiple testing using the false discovery rate method by Benjamini et al (1995).

S4. Independent confirmation of superior sequence stability of $coRPGR^{ORF15}$. The National Genetics Reference Laboratory (NGRL) in Manchester identified multiple potential mutations in the $wtRPGR^{ORF15}$ construct (top). There were six potential frame shift mutations (two deletions, four insertions) and 74 additional ambiguous base calls. In contrast, the sequence of the $coRPGR^{ORF15}$ construct was confirmed to be intact with at least two times coverage (bottom). The symbol # indicates use of reverse primers.

S5. Flow cytometric analysis of $\text{RPGR}^{\text{ORF15}}$ expression. (**A**) HEK293T cells were transfected with either $coRPGR^{ORF15}$ (coRPGR), $wtRPGR^{ORF15}$ (wtRPGR), or *eGFP* containing control plasmids (scale bar = 20µm). (**B**) Harvested cells were immuno-labelled with primary anti-RPGR/secondary fluorescent antibodies and *eGFP* cells were used to set the lower end of the FACS gating for fluorescence in the far-red range as they were incubated with secondary antibody only. Positive controls (naïve HEK293T cells exposed to rabbit anti-βactin and donkey anti-rabbit with conjugated Alexa-Fluor 635) were then used to define the upper end of the fluorescence gate setting. Cells transfected with the $coRPGR^{ORF15}$ construct (co) showed higher fluorescence intensity than the cells transfected with the wild-type construct (wt). The Shapiro-Wilk test rejected the null-hypothesis for normality of the data sets ($p < 0.05$) and the Kruskal Wallis non-parametric test demonstrated a robust statistical difference between the cohorts ($p < 0.01$, $n = 9$). Box plot (median, box delineates lower and upper quartile, whiskers minimum and maximum) of median fluorescence intensities in arbitrary units [AU].

S6. RPGR gene therapy shows no toxicity in electroretinography (ERG) of unilaterally treated wild-type mice. ERG recordings in *C57BL/6J* mice after unilateral subretinal injection of AAV.RK.coRPGR (red) vs. no treatment (black). (**A**) shows data at two months of age (PM2), (**B**) at PM4 and (**C**) at PM6, the last time point tested. Factorial ANOVA for repeated measures retained the null hypothesis (no difference) in all analyses. Lines indicate mean amplitudes ± 95% confidence interval (whiskers).

S7. RPGR gene therapy shows no toxicity in electroretinography (ERG) of bilaterally treated wild-type mice. ERG recordings in *C57BL/6J* mice after bilateral subretinal injection of AAV.RK.coRPGR (red) and AAV.control (black). (**A**) shows data at two months of age (PM2), (**B**) at PM4 and (**C**) at PM6, the last time point tested. Factorial ANOVA for repeated measures retained the null hypothesis (no difference) in all analyses. Lines indicate mean amplitudes ± 95% confidence interval (whiskers).

S8. Representative retinal images of *C57BL/6J* mice at postnatal month 6 (PM6). Two columns represent eyes of the treatment group (left) and sham control group (right). Using scanning laser ophthalmoscopy imaging in the infrared mode, the focal plane was set to inner retina (top row) or outer retina (middle row). Bottom row demonstrates the physiologically weak autofluorescence in wild-type mice.

S9. ERG recordings in *Rpgr*$^{-/y}$ mice after *coRPGR*^{ORF15} gene therapy. Top (**A-C**) shows data from unilateral trial; bottom (**D-F**) from bilateral trial. Mean amplitudes (± 95% confidence interval) are shown in red for treated eyes and black for untreated or sham treated eyes. (**A** and **D**) show data at two months of age (PM2), (*B* and *E*) at PM4 and (**C** and **F**) at PM6, the last time point tested. Treatment with AAV.RK.coRPGR led to significant improvement of dark adapted ERG amplitudes (left panel in **B-C**) in the unilateral treatment trial. The treatment effect in the light adapted b-wave amplitudes (right panel in **C**) only became apparent at PM6. Amplitudes in treated eyes were consistently higher then in the sham treated eyes (**D-F**), but this only reached significance at PM2 (**D**) and PM6 (**F**) for dark adapted responses and PM4 (**E**) for light adapted responses.

S10. ERG recordings in *C57BL/6J*$^{Rd9/Boc}$ mice after *coRPGR*^{ORF15} gene therapy. Top (**A-C**) shows data from unilateral trial; bottom (**D-F**) from bilateral trial. Mean amplitudes (± 95% confidence interval) are shown in red for treated eyes and black for untreated or sham treated eyes. (**A** and **D**) show data at two months of age (PM2), (**B** and **E**) at PM4 and (**C** and **F**) at PM6, the last time point tested. Treatment with AAV.RK.coRPGR led to significant improvement of dark adapted ERG amplitudes (left panel in **B-C**) in the unilateral treatment trial. There was not significant treatment effect in the light adapted amplitudes (right panels) or in dark- or light adapted responses in the bilateral trial. However, amplitudes in treated eyes were consistently higher then in the sham treated eyes without reaching significance levels in this phenotypically mild disease model.

Figure S1

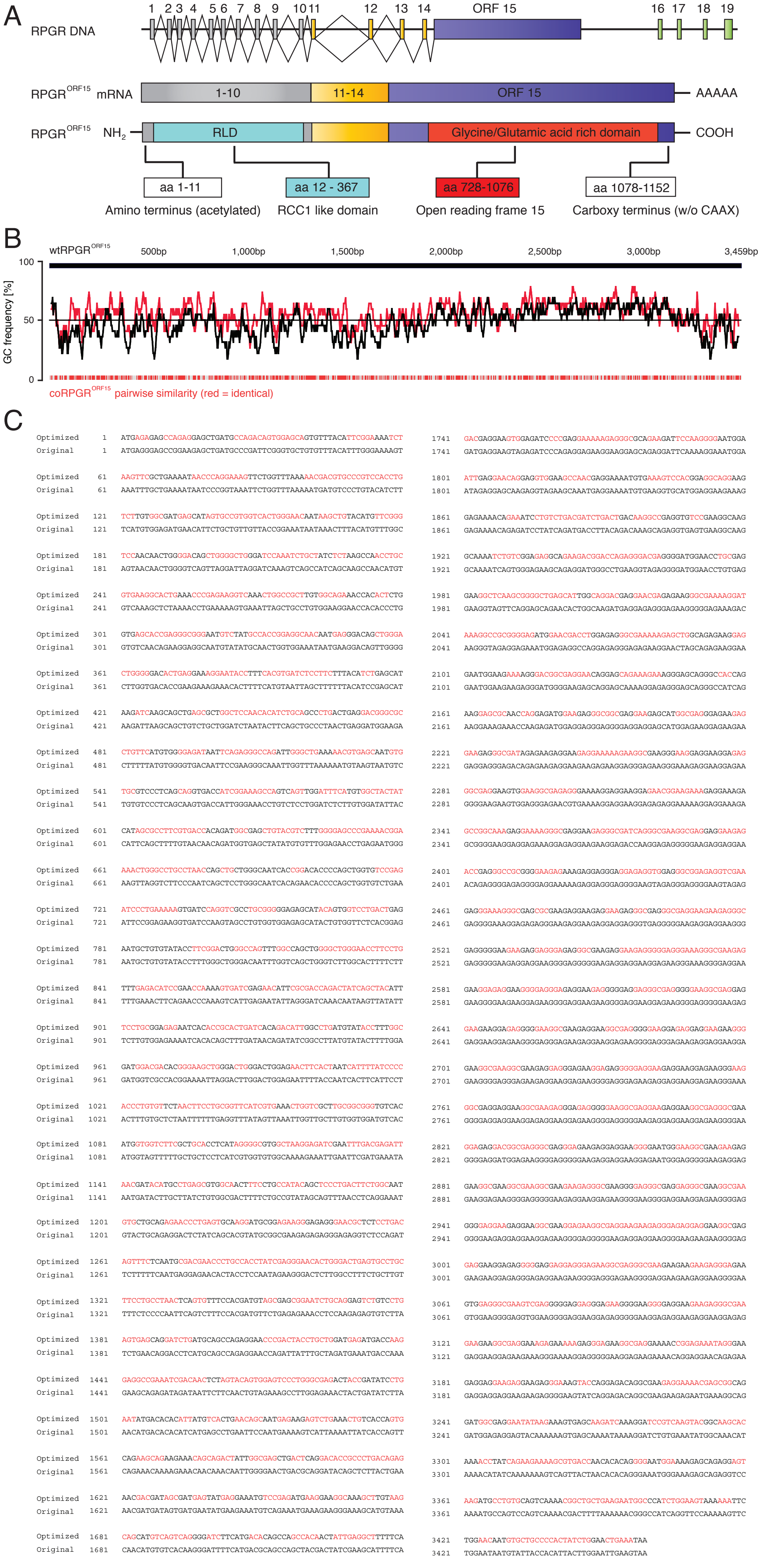

A
RPGR DNA
1 2 3 4 5 6 7 8 9 10 11 12 13 14 ORF 15 16 17 18 19
RPGR ORF15 mRNA
1-10
11-14
ORF 15
AAAAA
RPGR ORF15
NH2
RLD
Glycine/Glutamic acid rich domain
COOH
aa 1-11
aa 12 - 367
aa 728-1076
aa 1078-1152
Amino terminus (acetylated)
RCC1 like domain
Open reading frame 15
Carboxy terminus (w/o CAAX)
B
wtRPGR ORF15
500bp
1,000bp
1,500bp
2,000bp
2,500bp
3,000bp
3,459bp
100
50
0
GC frequency [%]
coRPGR ORF15 pairwise similarity (red = identical)
C
Optimized
Original

Figure S2

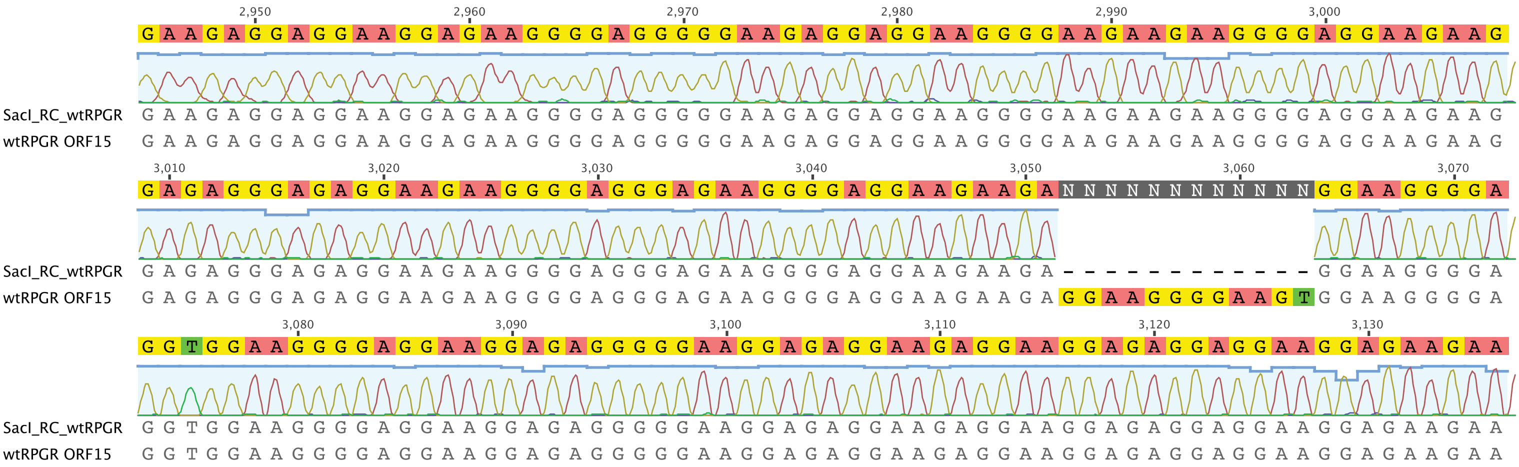

2,950
2,960
2,970
2,980
2,990
3,000
GAAGAGGAGGAAGGAGAAGGGGAGGGGGAAGAGGAGGAAGGGGAAGAAGAAGGGGAGGAAGAAG
SacI_RC_wtRPGR GAAGAGGAGGAAGGAGAAGGGGAGGGGGAAGAGGAGGAAGGGGAAGAAGAAGGGGAGGAAGAAG
wtRPGR ORF15 GAAGAGGAGGAAGGAGAAGGGGAGGGGGAAGAGGAGGAAGGGGAAGAAGAAGGGGAGGAAGAAG
3,010
3,020
3,030
3,040
3,050
3,060
3,070
GAGAGGGAGAGGAAGAAGGGGAGGGAGAAGGGGAGGAAGAAGANNNNNNNNNNNNGGAAGGGGA
SacI_RC_wtRPGR GAGAGGGAGAGGAAGAAGGGGAGGGAGAAGGGGAGGAAGAAGA------------GGAAGGGGA
wtRPGR ORF15 GAGAGGGAGAGGAAGAAGGGGAGGGAGAAGGGGAGGAAGAAGAGGAAGGGGAAGTGGAAGGGGA
3,080
3,090
3,100
3,110
3,120
3,130
GGTGGAAGGGGAGGAAGGAGAGGGGGAAGGAGAGGAAGAGGAAGGAGAGGAGGAAGGAGAAGAA
SacI_RC_wtRPGR GGTGGAAGGGGAGGAAGGAGAGGGGGAAGGAGAGGAAGAGGAAGGAGAGGAGGAAGGAGAAGAA
wtRPGR ORF15 GGTGGAAGGGGAGGAAGGAGAGGGGGAAGGAGAGGAAGAGGAAGGAGAGGAGGAAGGAGAAGAA

Figure S3

A

	wtRPGR^{ORF15}	*coRPGR^{ORF15}*
**deletions** [mean (range)]	1.5 (0 - 4)	*nil*
**insertions** [mean (range)]	0.5 (0 - 1)	*nil*
**point mutations** [mean (range)]	17.8 (9 - 33)	*nil*
**Total** [mean (range)]	19.75 (9 - 38)	*nil*

B

	wtRPGR^{ORF15}	*coRPGR^{ORF15}*	***p* - value** [FDR corrected]
**99% (Phred Q20)**	91.3 ± 1.0	96.6 ± 0.9	0.0005
**99.9% (Phred Q30)**	83.1 ± 2.6	90.5 ± 2.6	0.0044
**99.99% (Phred Q40)**	73.6 ± 3.8	82.3 ± 3.0	0.0054
**Confidence Mean**	49.1 ± 1.2	52.4 ± 1.0	0.0044
**Expected Errors**	82.9 ± 25.1	14.4 ± 5.1	0.0023

Figure S4

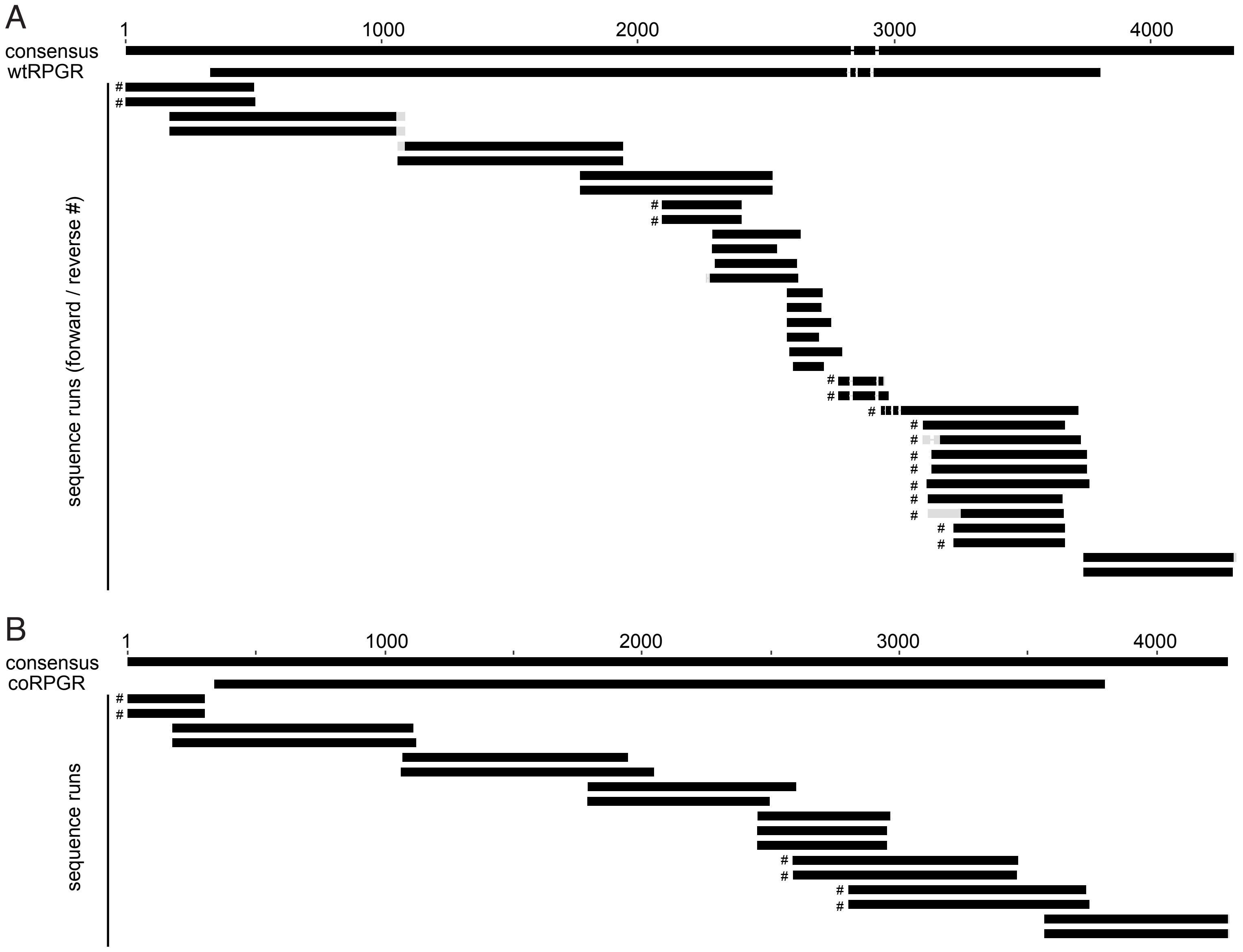
A
1
1000
2000
3000
4000
consensus
wtRPGR
sequence runs (forward / reverse #)
B
1
1000
2000
3000
4000
consensus
coRPGR
sequence runs

Figure S5

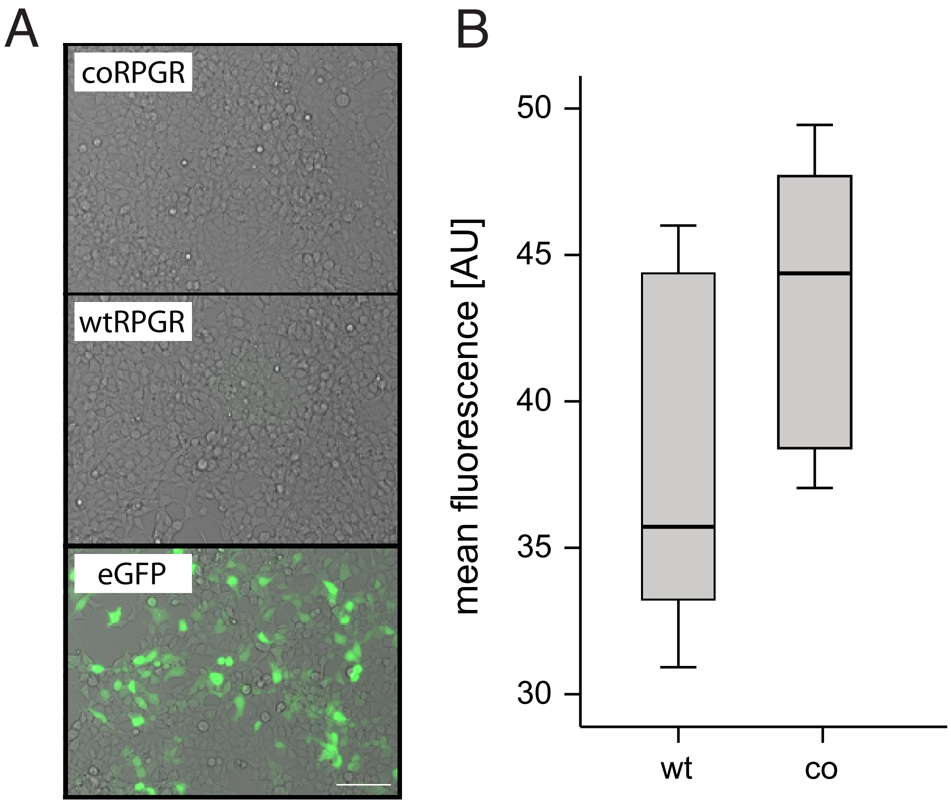
A
coRPGR
wtRPGR
eGFP
B
mean fluorescence [AU]
50
45
40
35
30
wt
co

Figure S6

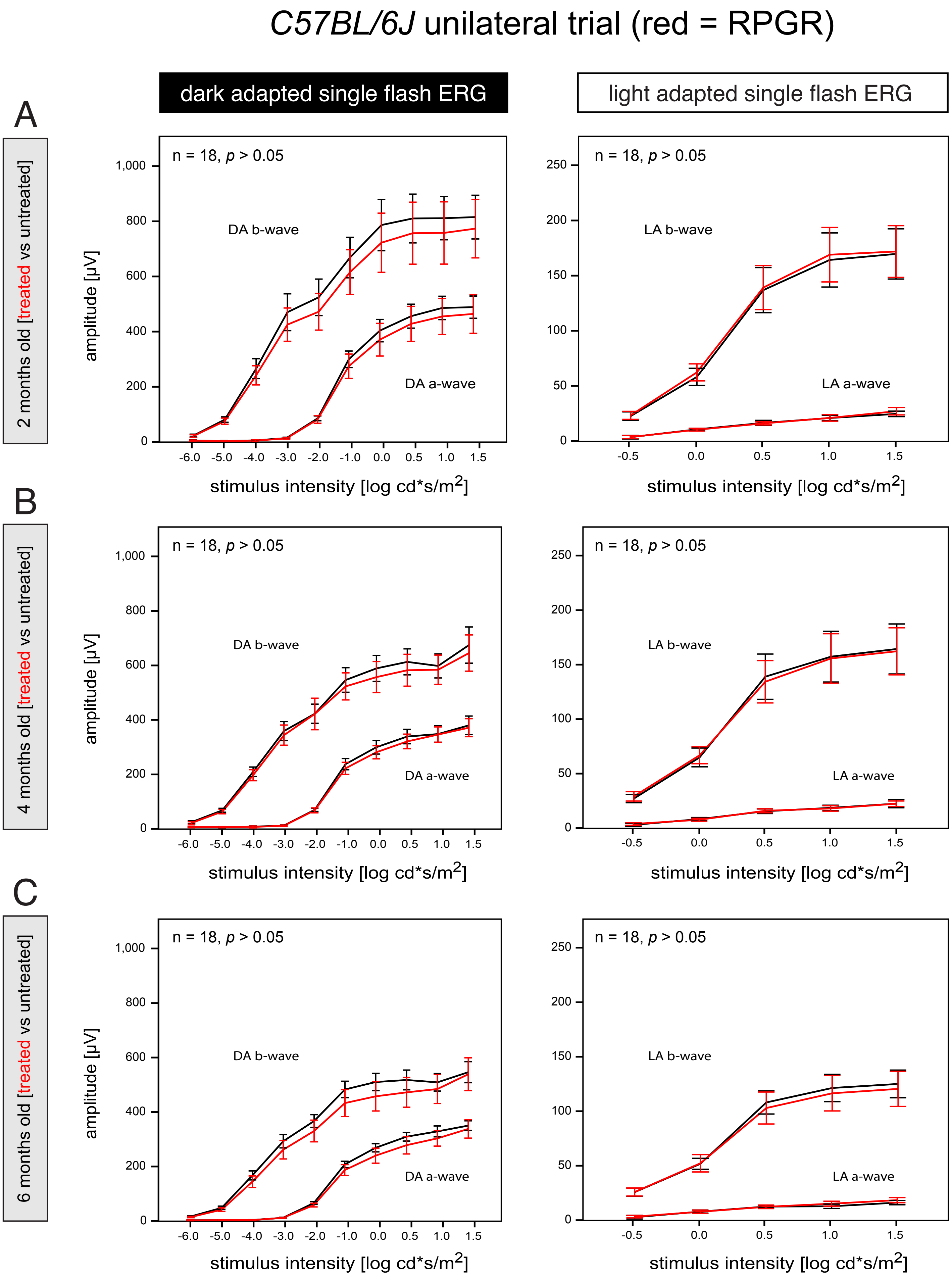

C57BL/6J unilateral trial (red = RPGR)
dark adapted single flash ERG
light adapted single flash ERG
A
2 months old [treated vs untreated]
n = 18, p > 0.05
DA b-wave
DA a-wave
LA b-wave
LA a-wave
amplitude [µV]
stimulus intensity [log cd*s/m2]
B
4 months old [treated vs untreated]
n = 18, p > 0.05
DA b-wave
DA a-wave
LA b-wave
LA a-wave
amplitude [µV]
stimulus intensity [log cd*s/m2]
C
6 months old [treated vs untreated]
n = 18, p > 0.05
DA b-wave
DA a-wave
LA b-wave
LA a-wave
amplitude [µV]
stimulus intensity [log cd*s/m2]

Figure S7

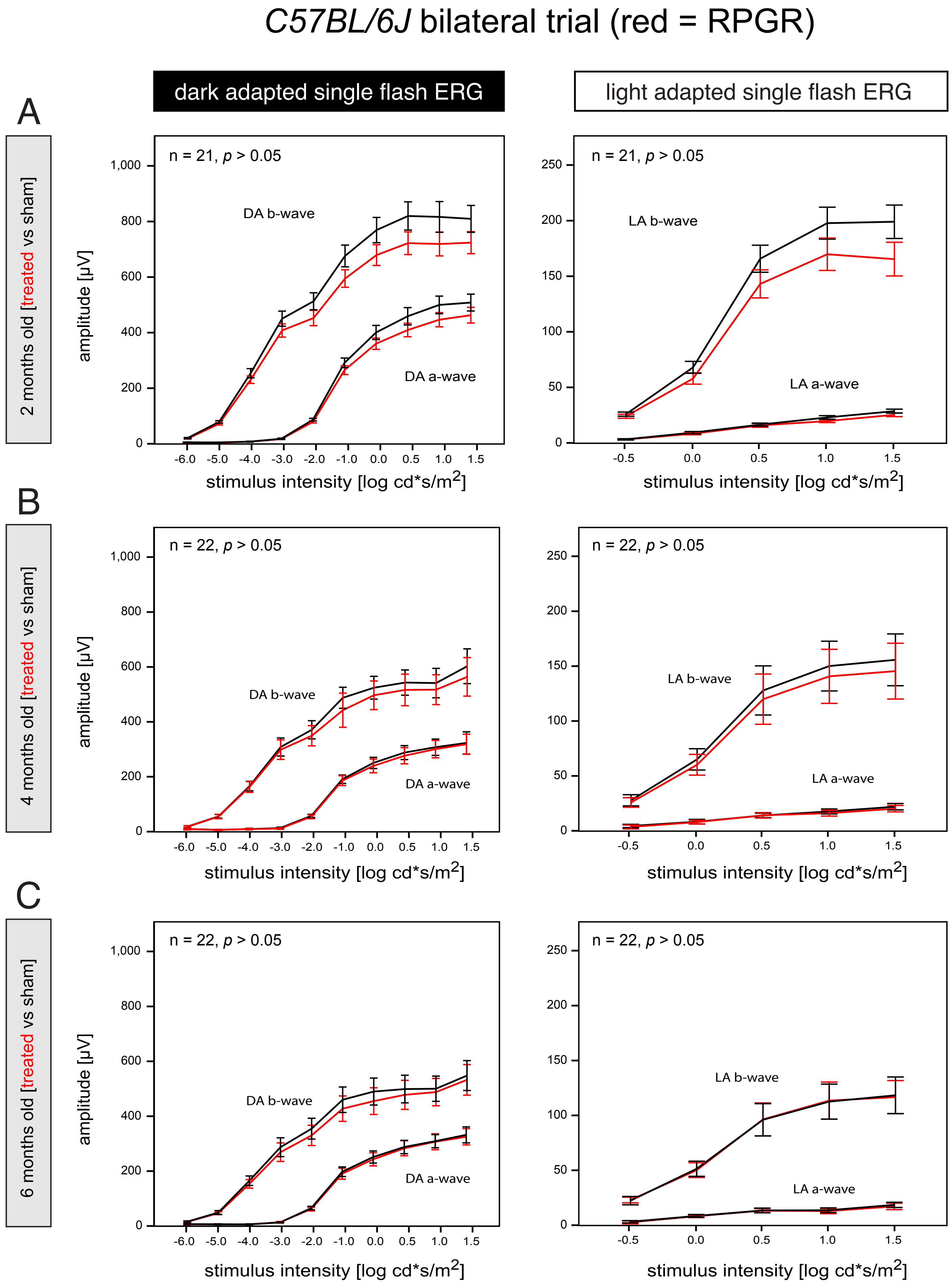

C57BL/6J bilateral trial (red = RPGR)
dark adapted single flash ERG
light adapted single flash ERG
A
2 months old [treated vs sham]
amplitude [µV]
n = 21, p > 0.05
DA b-wave
DA a-wave
LA b-wave
LA a-wave
stimulus intensity [log cd*s/m2]
B
4 months old [treated vs sham]
n = 22, p > 0.05
C
6 months old [treated vs sham]
n = 22, p > 0.05
0
200
400
600
800
1,000
50
100
150
250
-6.0
-5.0
-4.0
-3.0
-2.0
-1.0
0.0
0.5
1.0
1.5
-0.5

Figure S8

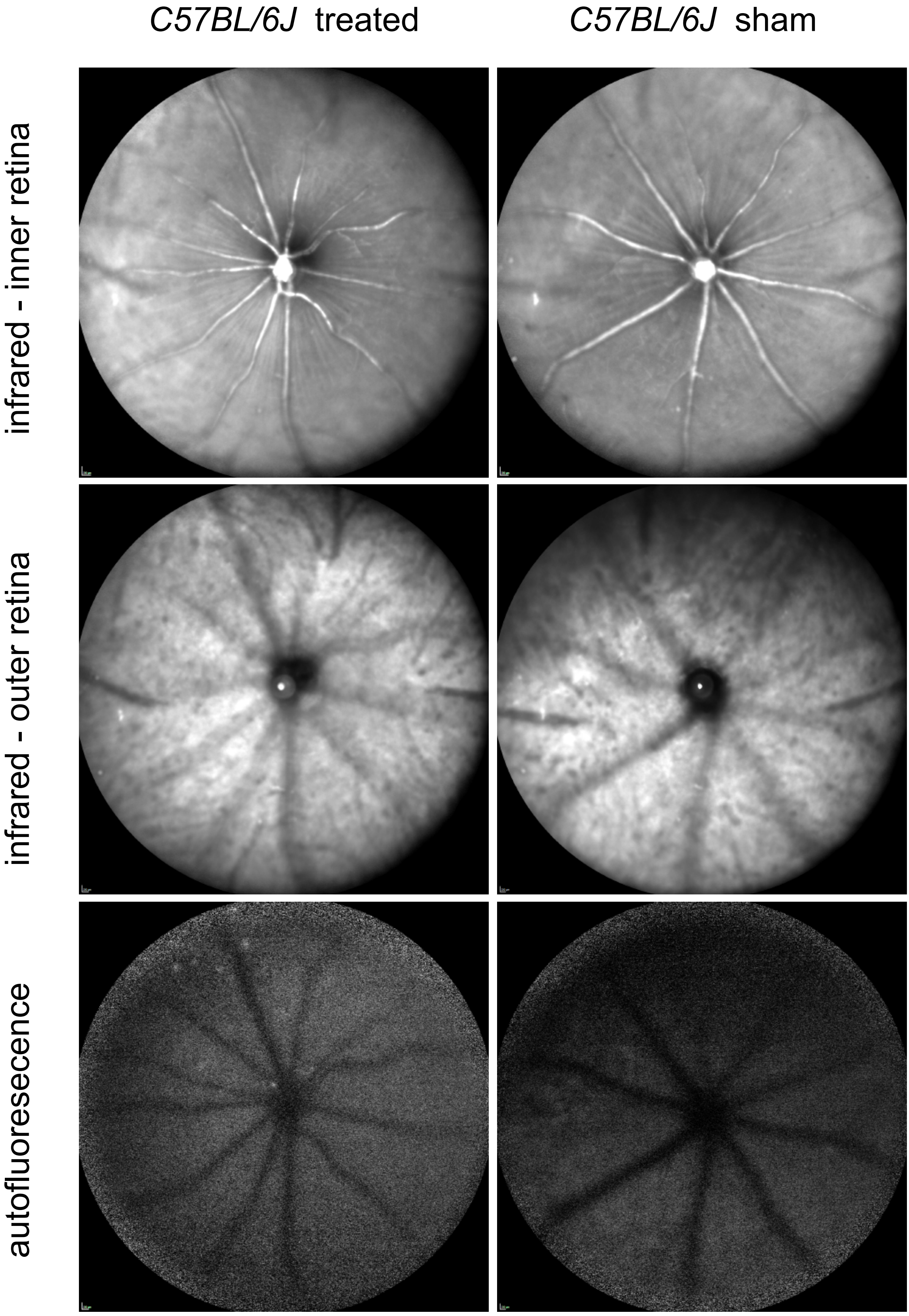

C57BL/6J treated
C57BL/6J sham
infrared - inner retina
infrared - outer retina
autofluoresecence

Figure S9

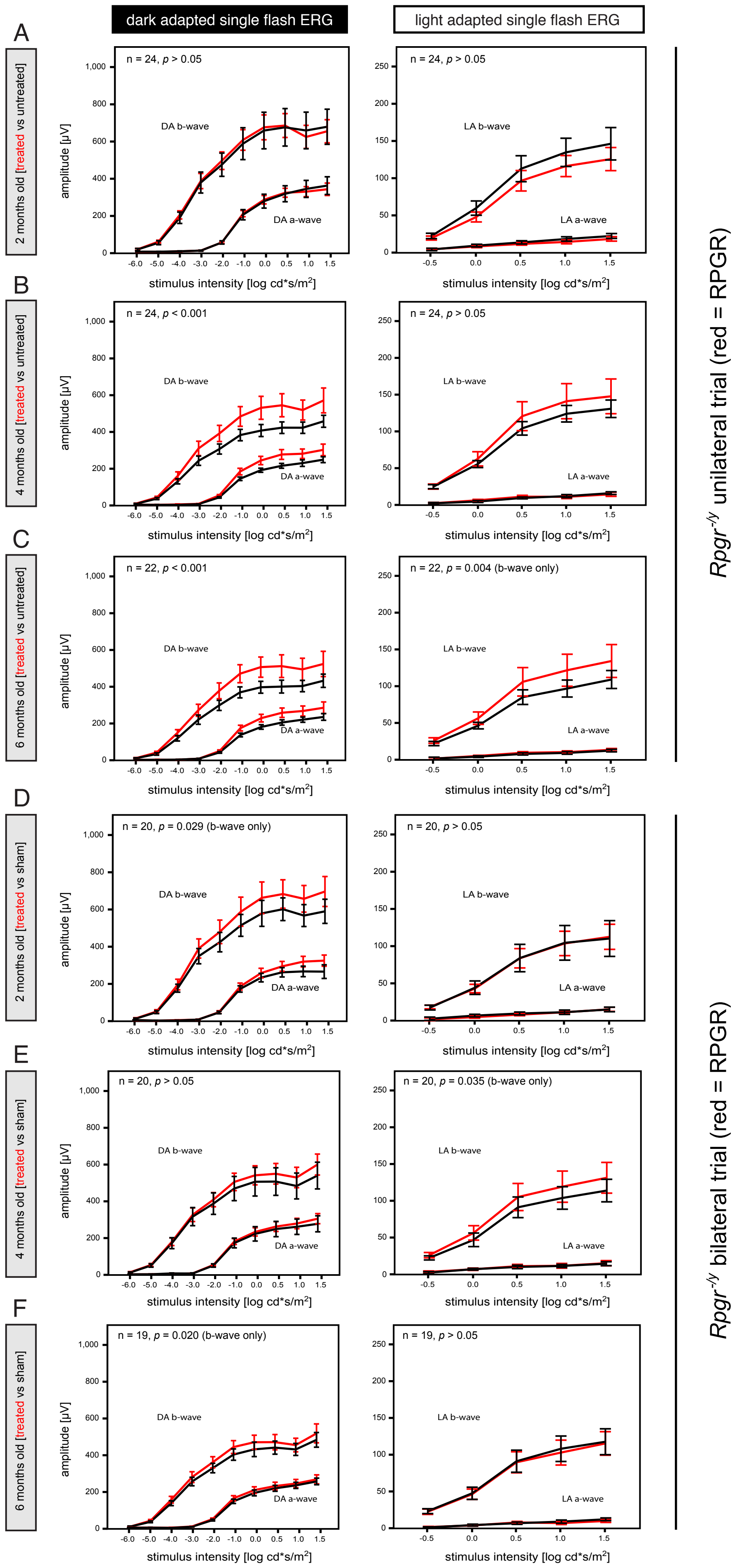
dark adapted single flash ERG
light adapted single flash ERG
A
2 months old [treated vs untreated]
n = 24, p > 0.05
DA b-wave
DA a-wave
LA b-wave
LA a-wave
amplitude [µV]
stimulus intensity [log cd*s/m²]
B
4 months old [treated vs untreated]
n = 24, p < 0.001
n = 24, p > 0.05
C
6 months old [treated vs untreated]
n = 22, p < 0.001
n = 22, p = 0.004 (b-wave only)
Rpgr-/y unilateral trial (red = RPGR)
D
2 months old [treated vs sham]
n = 20, p = 0.029 (b-wave only)
n = 20, p > 0.05
E
4 months old [treated vs sham]
n = 20, p > 0.05
n = 20, p = 0.035 (b-wave only)
F
6 months old [treated vs sham]
n = 19, p = 0.020 (b-wave only)
n = 19, p > 0.05
Rpgr-/y bilateral trial (red = RPGR)

Figure S10

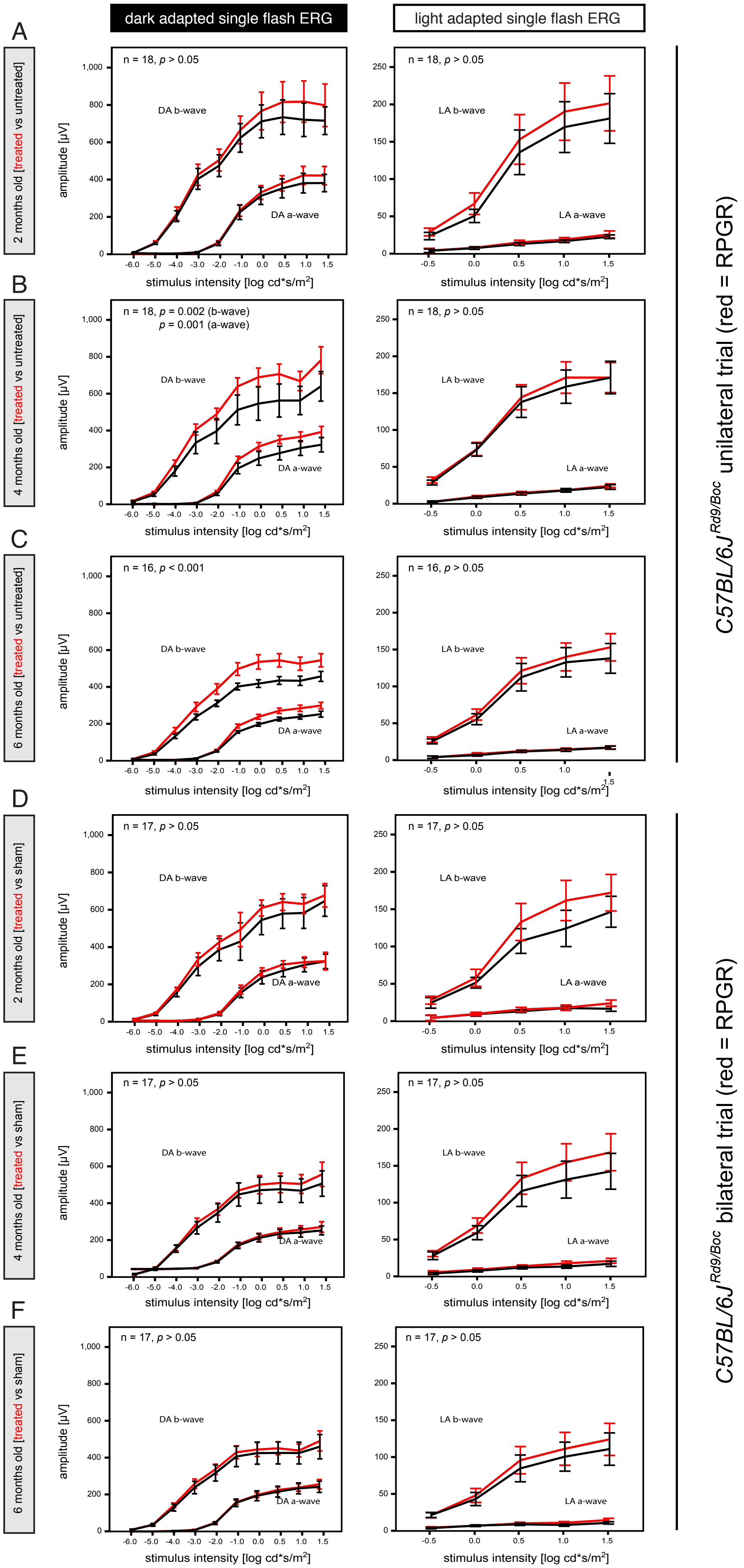
dark adapted single flash ERG
light adapted single flash ERG
A
2 months old [treated vs untreated]
n = 18, p > 0.05
DA b-wave
DA a-wave
LA b-wave
LA a-wave
B
4 months old [treated vs untreated]
n = 18, p = 0.002 (b-wave)
p = 0.001 (a-wave)
n = 18, p > 0.05
C
6 months old [treated vs untreated]
n = 16, p < 0.001
n = 16, p > 0.05
D
2 months old [treated vs sham]
n = 17, p > 0.05
E
4 months old [treated vs sham]
F
6 months old [treated vs sham]
amplitude [µV]
stimulus intensity [log cd*s/m2]
C57BL/6JRd9/Boc unilateral trial (red = RPGR)
C57BL/6JRd9/Boc bilateral trial (red = RPGR)
